# Annotations

**Published:** 1909-02-27

**Authors:** 


### ANNOTATIONS.

### The Director of the Radium Institute.

The Polyclinic, as is generally recognised, owed more probably to its medical superintendent, Captain Hayward Pinch, than to almost any single person. For ten years he has rendered loyal and zealous service as lecturer and teacher, and under his able superintendence the work of the College has been organised on the best and most practical lines to meet the requirements of the graduates who have attended its courses. We are glad to note that the Council of the Polyclinic have placed on record their high and cordial appreciation of Captain Pinch, who, they justly declare, has devoted himself without qualification or stint to the interests of the Polyclinic during the years he has been intimately associated with its work. We have frequently heard the most grateful encomiums passed upon Captain Pinch by members of and subscribers to the Polyclinic who, without exception, recognise the thoroughness of Captain Pinch's work, and the courtesy and good-will which have marked his relations with all the friends of the College. Captain Pinch has accepted the office of director of the Radium Institute, and is about to proceed abroad in connection with the duties of his office. The organisation of the Radium Institute will afford a unique opportunity for the development of Captain Pinch's organising powers. His selection for the post of director marks the good judgment and energy which the promoters of the Institute are displaying in the performance of a difficult task, and must secure for its work the fullest opportunity for successful development. This appointment will give satisfaction to the medical profession, and should win public confidence. We congratulate Captain Pinch most heartily; he has thoroughly deserved his promotion and we wish him success in the most interesting and novel field of labour on which he is about to enter.

### Medical Defence in Scotland.

The sixth annual report of the Medical and Dental Defence Union of Scotland demonstrates clearly that this organisation is emerging from the difficult and precarious stage, through which all such associations have to pass in their infancy, into maturity and assured prosperity. The membership now stands at 1,177, of whom 240 have joined during the past year. The invested funds have reached the respectable figure of £1,200. On the enormous advantage which these corporate defence agencies afford to medical men in general comment was offered in these columns last year (The Hospital, June 27, 1908, p. 335). The only point which suggests itself for discussion is the advisability of multiplying such societies as compared with the strengthening of the most powerful of those already in existence. Now, however advantageous concentration may be, it is to be remembered that Scotland has its own laws, and that the medical profession in that country is quite naturally disposed to assert its independence, though it is just as much, or just as little, under the tutelage of the General Medical Council as in the rest of the Kingdom. The contention must, we think, be admitted that a council sitting in Scotland is more competent to deal with Scottish difficulties than one which sits in some other part of the United Kingdom. There is, moreover, one point on which our English defence societies might find it worth while to take a hint from the Scottish Union, and that is by issuing, as the latter does, printed forms of indemnity to be granted by friends of patients in respect of lunacy certificates, and of agreement for use between practitioner and assistant. Before leaving this topic we may quite briefly note that defence unions, modelled more or less upon those with which we in this country are familiar, have been formed in some 12 or 13 of the States of the Union in America, the latest addition to the number being in Kentucky.

### School Dentistry at Cambridge.

The medical profession awoke some considerable number of years ago to the very widespread prevalence of dental caries among all classes of the population, but especially among the poor, and to its disastrous effects on the national health and physique. Year in year out has the medical (and, of course, the dental) Press urged the necessity that exists for proper supervision of the teeth of the nation's children as a vital part of any scheme of hygiene. To do the well-educated laity justice they have hearkened to these counsels thoroughly as far as their own children are concerned; there is admittedly far less caries and pyorrhœa among the upper and middle classes than there was ten or fifteen years ago. But the poor are just as bad as ever; and unless they are taken in hand at an early age, when the mischief is beginning and the mind impressionable, it is difficult to see much prospect of improvement. What can be done by careful supervision, and how essential it is that something should be done, anyone may see who will read the report of Dr. Cunningham and Mr. Gant on the first eighteen months' work of the latter among the school children of Cambridge. This work, we hasten to add, was remunerated by private generosity, not from public funds. At thirteen years of age the average number of unsaveable teeth per child was two, and of carious but still saveable ones an additional seven. This, be it noted, at an age when the second dentition is not yet complete, and when several of the teeth are almost brand new. Out of 2,522 children examined 617 were made artificially sound by having all their carious (permanent) teeth stopped, while the same was done for the others except for the hopeless teeth which had got beyond all chance of redemption. Caries was found commencing in the permanent set as early as the sixth year, and at seven quite a number of children already presented unsaveable teeth. This truly awful state of affairs is a disgrace to the nation, on the removal of which the medical profession must insist until the question is taken up by the proper authorities.

